# Functional evaluation using the modified Japanese Orthopedic Association score (mJOA) for cervical spondylotic myelopathy disease by age, gender, and type of disease

**Published:** 2012-11

**Authors:** Parisa Azimi, Sohrab Shahzadi, Edward C. Benzel, Ali Montazari

**Affiliations:** ^*a*^Department of Neurosurgery, Shahid Beheshti University of Medical Sciences, Tehran, Iran.; ^*b*^Cleveland Clinic Foundation, Department of Neurosurgery, Cleveland, Ohio, USA.; ^*c*^Mental Health Research Group, Health Metrics Research Centre, Iranian Institute for Health Sciences Research, ACECR, Tehran, Iran.

## Abstract

**Background:**

Cervical spondylotic myelopathy (CSM) is a common cause of significant clinical morbidity. The modified Japanese Orthopedic Association score (mJOA) is a very short instrument for functional evaluation in these patients. This study aimed to evaluate functionality in patients diagnosed with CSM diseases using the mJOA and to compare the scores based on age, sex, and type of disease.

**Methods:**

A sample of patients with CSM who were candidate for decompressive surgery entered into this cross sectional study. The mJOA scores were obtained for the functional assessment of patients. Score on the mJOA range from 0 to 18 with higher scores indicating a better condition. In addition to descriptive statistics, the data were compared among study subgroups as categorized by age, gender and type of disease.

**Results:**

A total of 63 patients were studied. The mean age of the patients was 54 ± 8.3 (SD)(range 21-79 years) All patients diagnosed as having cervical herniated disc (n = 36) or cervical spinal stenosis (n = 27). Overall, the mean mJOA score for all patients was 9.82 (SD = 1.0). The mJOA score for men were significantly higher than women (P less than 0.0001). The mJOA score in younger patients was significantly higher than older patients (P less than 0.0001). No significant difference was observed for type of disease (P = 0.47).

**Conclusions:**

The findings of the study suggest that functionality score as measured by the mJOA can be a useful parameter for clinicians help them to manage patients with cervical spondylotic myelopathy prior to surgery.

**Keywords:**

Score, Cervical, Spondylotic, Myelopathy


					Volume 4, Suppl. 1 Nov 2012
Publisher: Kermanshah University of Medical Sciences
URL: http://www.jivresearch.org
Copyright:
Congress of Iranian Neurosurgeons, 14-16 November 2012, Kermanshah, Iran
Paper No. 42
Functional evaluation using the modified Japanese Orthopedic
Association score (mJOA) for cervical spondylotic myelopathy
disease by age, gender, and type of disease
Parisa Azimi a,*
, Sohrab Shahzadi a
, Edward C. Benzel b
, Ali Montazari c
a
Department of Neurosurgery, Shahid Beheshti University of Medical Sciences, Tehran, Iran.
b
Cleveland Clinic Foundation, Department of Neurosurgery, Cleveland, Ohio, USA.
c
Mental Health Research Group, Health Metrics Research Centre, Iranian Institute for Health Sciences Research, ACECR,
Tehran, Iran.
Abstract:
Background: Cervical spondylotic myelopathy (CSM) is a common cause of significant clinical morbidity. The
modified Japanese Orthopedic Association score (mJOA) is a very short instrument for functional evaluation in these
patients. This study aimed to evaluate functionality in patients diagnosed with CSM diseases using the mJOA and to
compare the scores based on age, sex, and type of disease.
Methods: A sample of patients with CSM who were candidate for decompressive surgery entered into this cross
sectional study. The mJOA scores were obtained for the functional assessment of patients. Score on the mJOA range
from 0 to 18 with higher scores indicating a better condition. In addition to descriptive statistics, the data were
compared among study subgroups as categorized by age, gender and type of disease.
Results: A total of 63 patients were studied. The mean age of the patients was 54  8.3 (SD)(range 21-79 years)
All patients diagnosed as having cervical herniated disc (n = 36) or cervical spinal stenosis (n = 27). Overall, the
mean mJOA score for all patients was 9.82 (SD = 1.0). The mJOA score for men were significantly higher than
women (P less than 0.0001). The mJOA score in younger patients was significantly higher than older patients (P less
than 0.0001). No significant difference was observed for type of disease (P = 0.47).
Conclusions: The findings of the study suggest that functionality score as measured by the mJOA can be a useful
parameter for clinicians help them to manage patients with cervical spondylotic myelopathy prior to surgery.
Keywords:
Score, Cervical, Spondylotic, Myelopathy
* Corresponding Author at:
Parisa Azimi: Department of Neurosurgery, Imam-Hossain Hospital, Shahid-Beheshti University of Medical Sciences, Imam-Hossain sq., Tehran, Iran.
Email: Parisa.azimi@gmail.com, (Azimi P.).

				

